# Supplementary material for: Quantifying biomolecular organisation in membranes with brightness-transit statistics
Source: Nat Commun. 2024 Aug 17;15:7082. doi: 10.1038/s41467-024-51435-1 (PMC11329664; doi:10.1038/s41467-024-51435-1)
Supplement: Supplementary file 1 — Supplementary Information [file 41467_2024_51435_MOESM1_ESM.pdf]

Supplementary Information

Quantifying biomolecular organisation in membranes with brightness-transit statistics

Falk Schneider[1,2*], Pablo F. Cespedes[1], Narain Karedla[1,3], Michael L. Dustin[1], Marco Fritzsche[1,3*]

[1]Kennedy Institute for Rheumatology, Roosevelt Drive, University of Oxford, Oxford, OX3 7LF, United Kingdom.

[2]Translational Imaging Center, University of Southern California, Los Angeles, California 90089, United States of America.

[3]Rosalind Franklin Institute, Harwell Campus, Didcot, OX11 0FA, United Kingdom.

*Correspondence to: falkschn@usc.edu, marco.fritzsche@kennedy.ox.ac.uk

Keywords:

Membrane, dynamics, FCS, FFS, scanning FCS, immune cells, oligomerisation, CD40

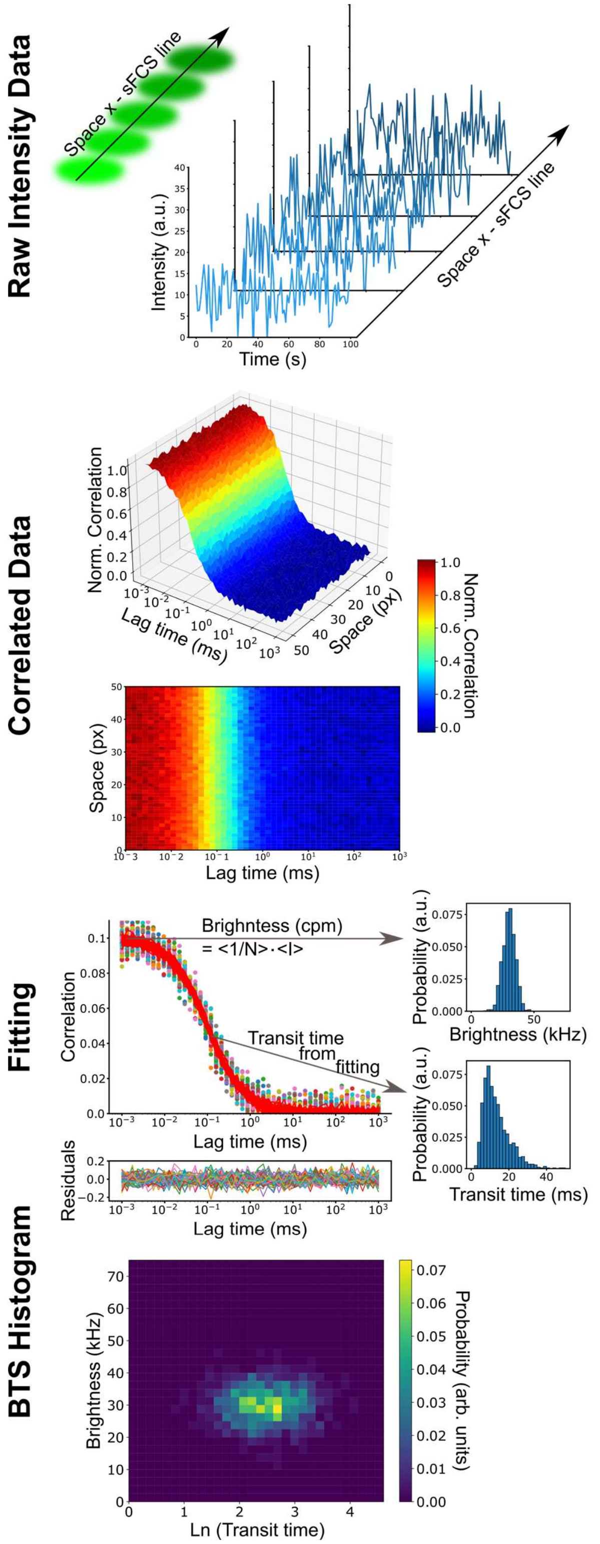

Intensity carpet
Acquire intensity fluctuation per pixel over time
xt-carpet aka intensity carpet

Notes & tips:
Scanning frequency determines temporal resolution
Increase acquisition time to improve SNR
Measure excitation laser power and keep it constant
Optimize acquisition parameters to keep photo-bleaching minimal

Temporally autocorrelate the intensity traces for every pixel

Correlation curves mapped across space

Notes & tips:
Intensity data can be cropped and corrected for photo-bleaching removing artefacts in the correlation data
Keep post-processing to a minium if possible and consistent across samples

Correlation carpet is a simplified and normalized representation of the 3D correlation curve array

Extract correlation curves for model fitting

Single correlation curves, fits and residuals
Fit data to appropriate model (e.g, 2D free)

Notes & tips:
Keep floating parameters to a minimum
Systematic deviations from 0 of the residuals indicate usage of a wrong model

Large number of curves from only a few sFCS acuqisitions allows statistical analysis of fitting parameters **by histogramming**

BTS histogram
Incorporate transit time and brightness (cpm) from the fitted data
Ln (Transit time) is normally distributed for free diffusion
Brightness is calculated from correlation amplitude (1/N)·average intensity of the pixel (normally distributed for monomer)

Supplementary Figure 1: BTS acquisition and analysis workflow. Data are acquired in the fashion of sFCS experiments, meaning acquisition of xt intensity carpets, kymographs. The intensity fluctuations are then correlated for every pixel yielding an autocorrelation curve for every pixel that can be fitted. The resulting transit time and brightness are plotted as 2D histogram to create the BTS histogram. This decouples the dynamic information from the spatial information and allows for unbiased statistical analysis.

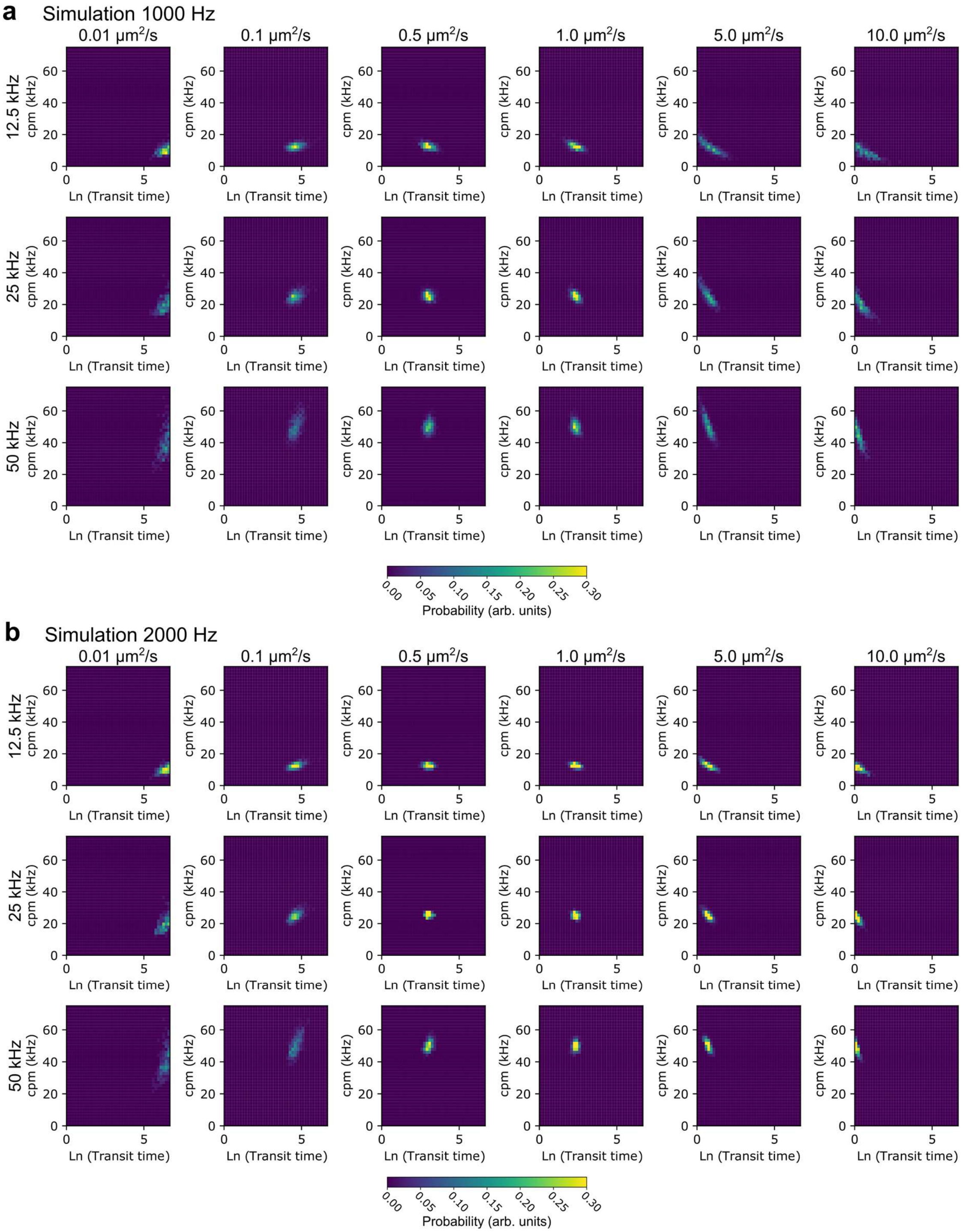

Supplementary Figure 2: sFCS simulations of free diffusion sampled at 1000 Hz and 2000 Hz. sFCS BTS diagrams for the full set of the simulations presented in Figure 1 main text. Freely diffusing molecules were randomly distributed in an 8 by 5 μm^2 box and their Brownian motion sampled by sFCS at 1000 Hz (a) or 2000 Hz (b) scanning frequency. 10 measurements, 500 curves were simulated per condition. Molecular brightness and diffusion coefficient were varied as indicated.

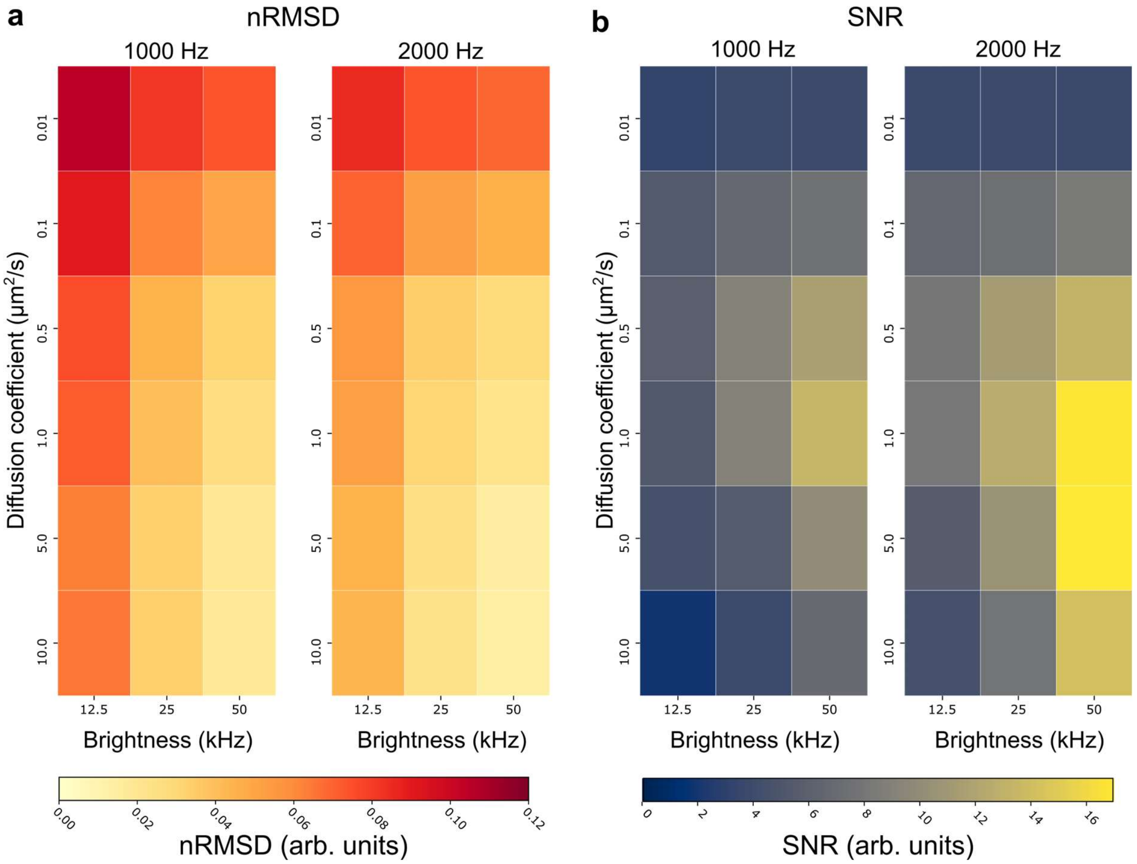

Supplementary Figure 3: Evaluation of sFCS data quality as function of diffusion coefficient and brightness (cpm). a nRMSD values (low values indicate good data quality) and **b** SNR (high values indicate good data quality). nRMSD values were calculated from the difference between the data and the fitted model basically quantifying mis-fit. The SNR values were calculated from the standard deviation of the auto-correlation (no fitting involved).

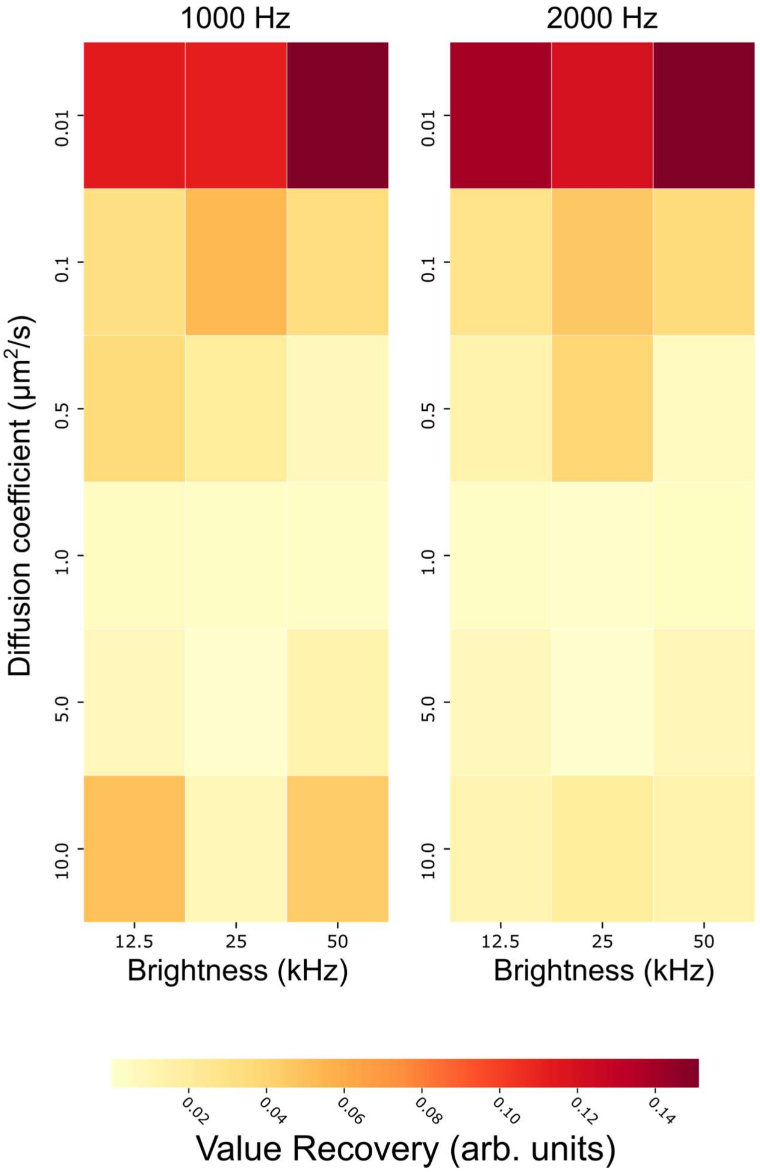

Supplementary Figure 4: Value Recovery. Recovered values (as median value of the whole set of data) were compared with input diffusion coefficients. Value Recovery is defined as $(|D_{input} - D_{output}|)/D_{input}$, with D_{input} as simulation input diffusion coefficient and D_{output} as the respective recovered diffusion coefficient. Values closer to zero indicate a more accurate value recovery which can also be thought of as a normalised error.

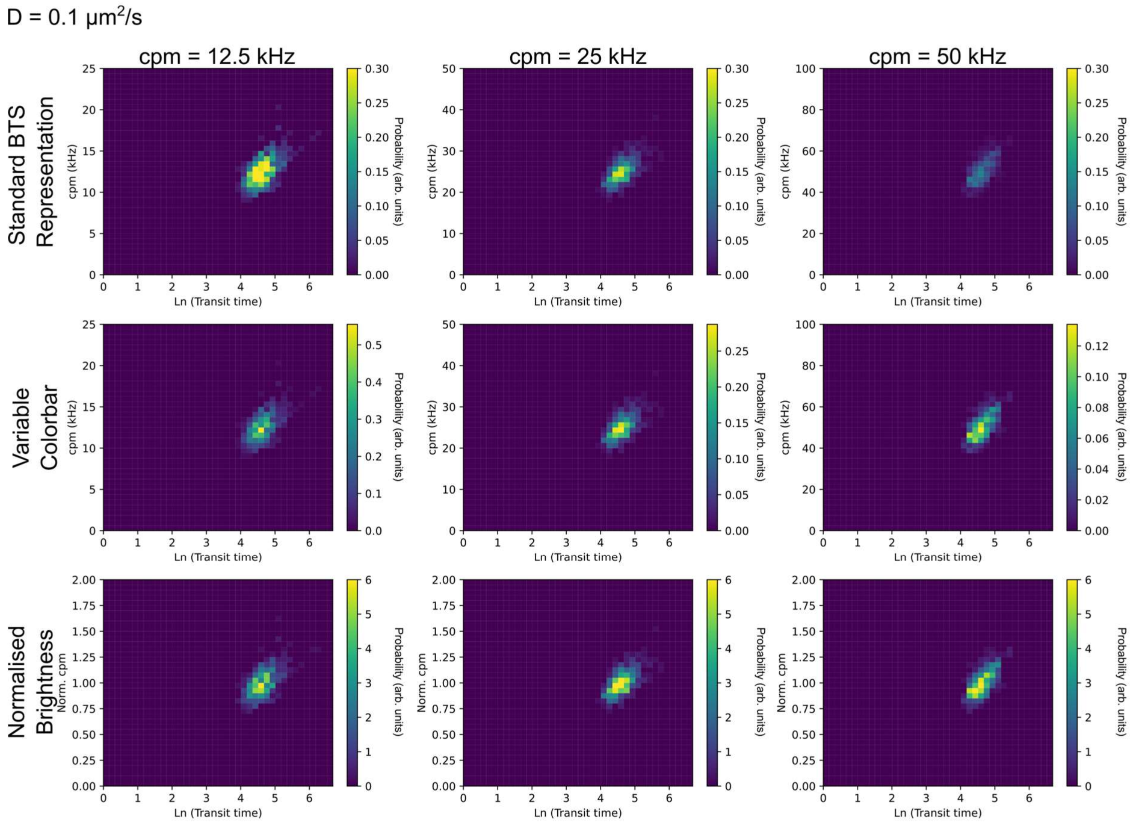

Supplementary Figure 5: BTS histogram normalization exemplified for simulated data with a diffusion coefficient of 0.1 µm^2/s sampled at 2000 Hz. Top column presents the data as in Figure 1c,d (and as in the rest of the manuscript). Middle column uses variable colour bars. Bottom column presents normalised data (cpm divided by mean cpm of condition).

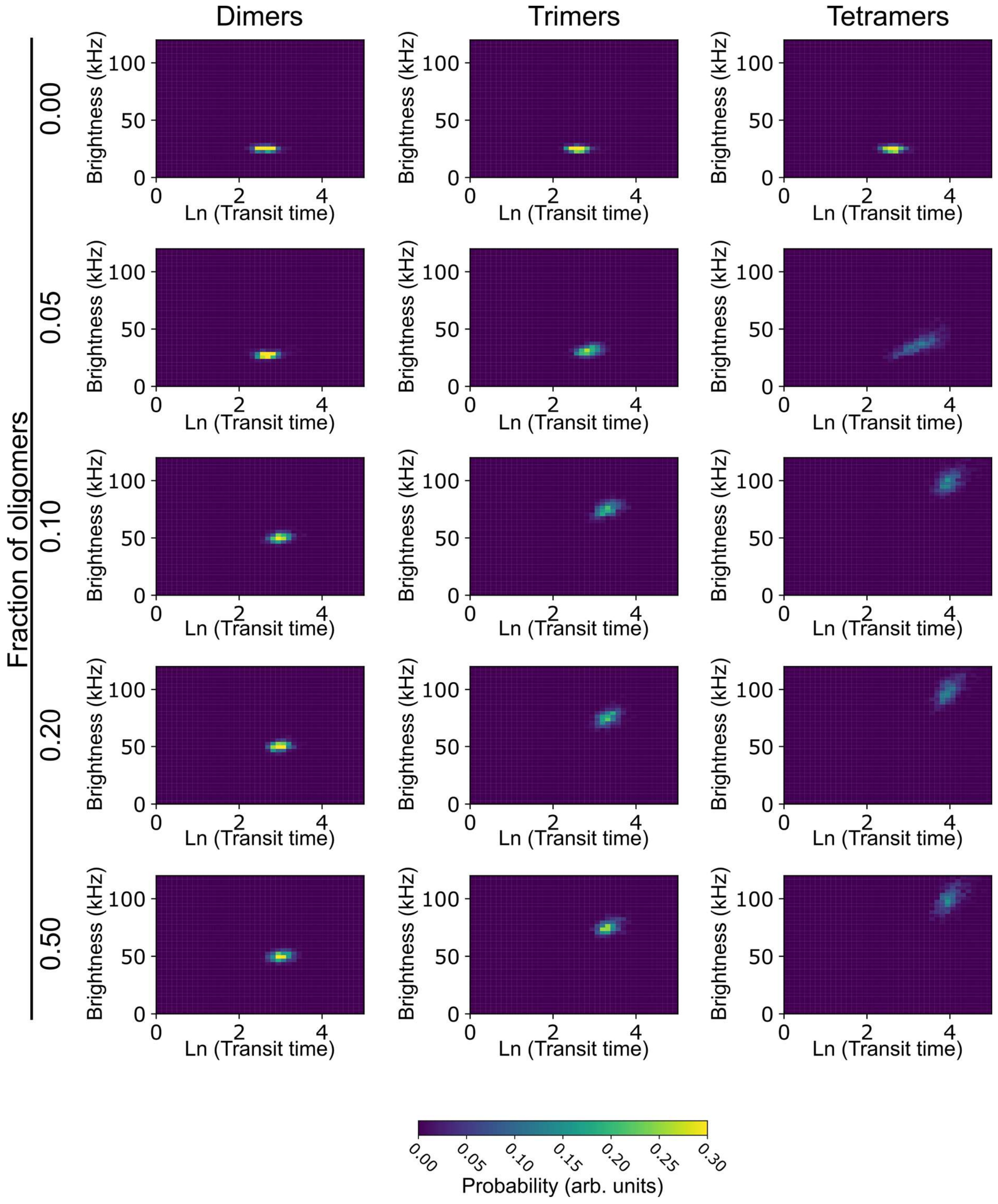

Supplementary Figure 6: BTS diagrams for the full range of oligomerisation simulations. In support of the simulation data on oligomerisation in Figure 1 all 2D BTS histograms are presented. The fraction of oligomers is varied as indicated. The total number of molecules in the simulation was kept constant. For every condition 10 measurements, 500 curves were simulated.

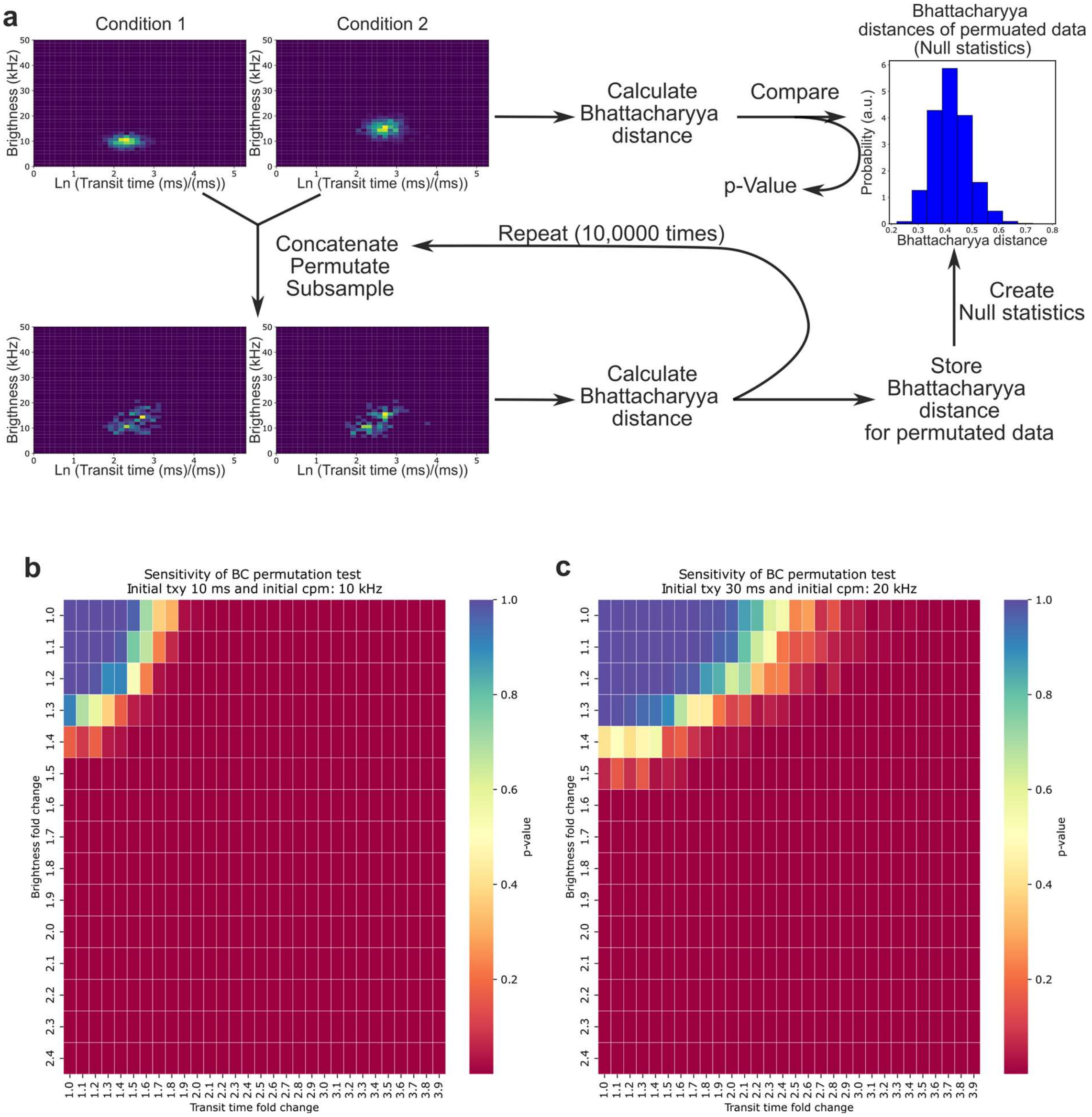

Supplementary Figure 7: Strategy to statistically compare BTS histograms and calculate p-Values. a Workflow for the comparison of two BTS histograms for hypothetical condition 1 and condition 2. **b,c** Sensitivity analysis using synthetic data (normally distributed brightness values and lognormally distributed transit time values). **b** initial parameter is 10 ms, 10 kHz and **c** 30 ms 20 kHz. Variance for transit time 0.1 and variance for brightness 0.15 in both cases.

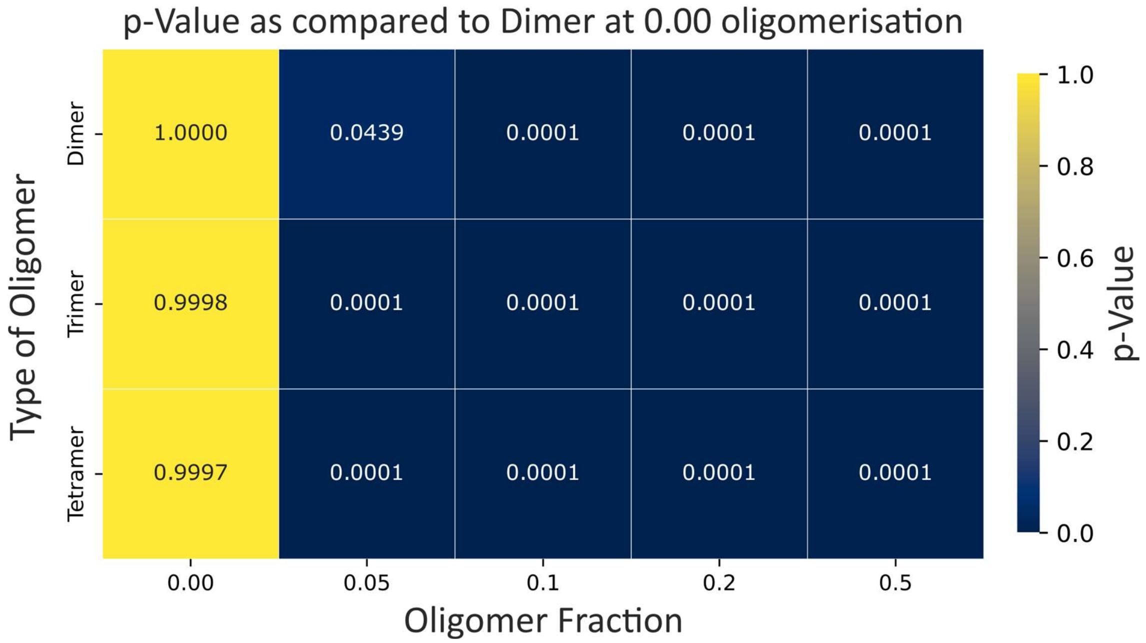

Supplementary Figure 8: Comparing BTS histograms from simulations on oligomerisation. BTS histograms were obtained from fitting simulated data. The BTS histograms were systematically compared using a permutation test (see Supplementary Figure 7). All BTS histograms were compared to the dimer control condition (i.e., a monomeric control, top left corner of the heatmap). High p-values indicate that the data are likely to originate from the same underlying 2D-distribution, low p-values (< 0.01) indicate that they are likely not originating from the same underlying 2D-distribution (i.e., significantly different). The first column essentially compares monomer condition from different simulation runs and shows that all BTS histograms are likely to originate from the same underlying distribution (as expected, all data are monomeric control).

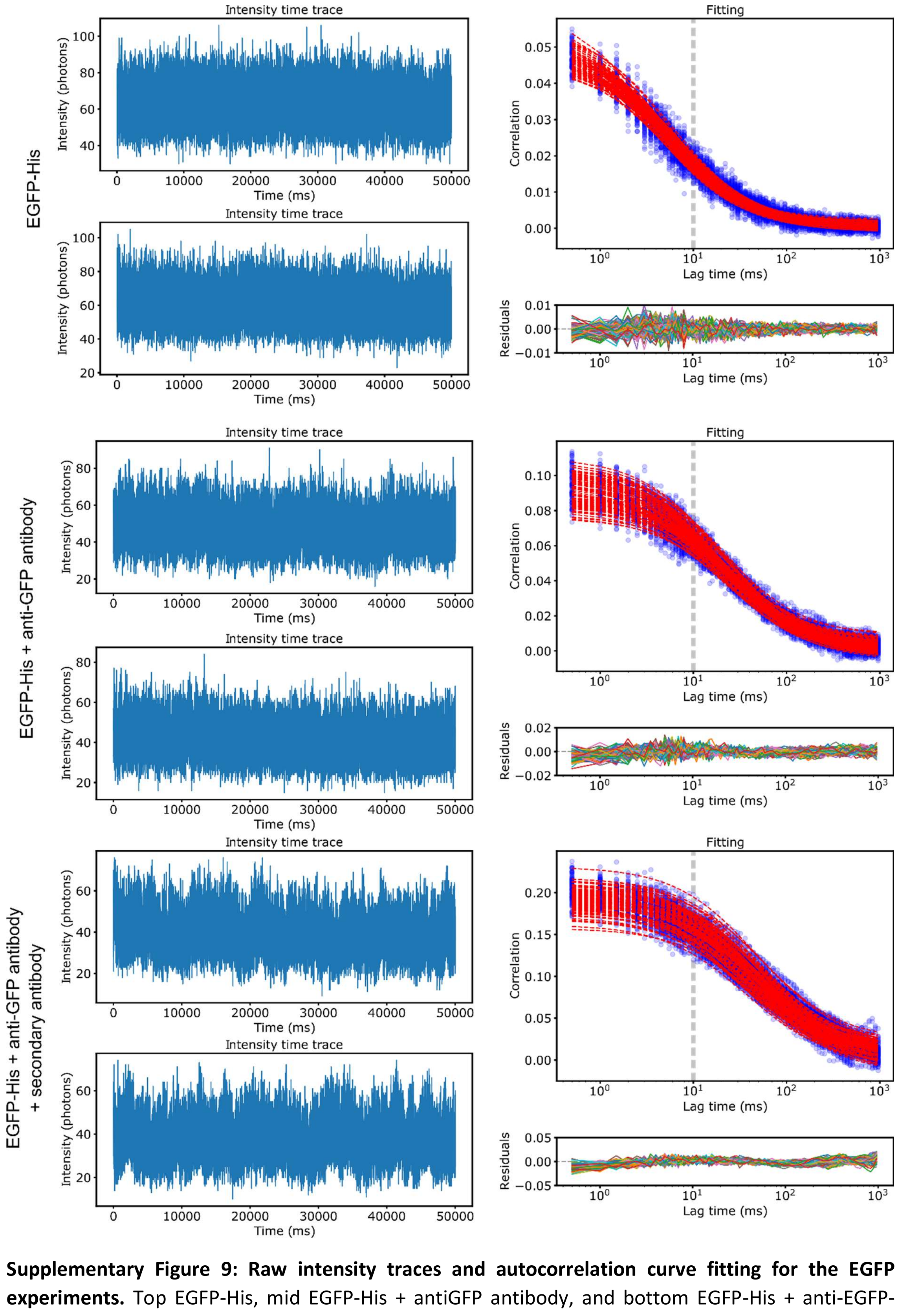

Supplementary Figure 9: Raw intensity traces and autocorrelation curve fitting for the EGFP experiments. Top EGFP-His, mid EGFP-His + antiGFP antibody, and bottom EGFP-His + anti-EGFP-antibody + secondary antibody. The three conditions show two representative sFCS measurements of

52 pixels each as intensity traces and fitting all resulting autocorrelation curves per pixel with a 2D diffusion model. Dashed line at 10 ms lag time was plotted for guidance.

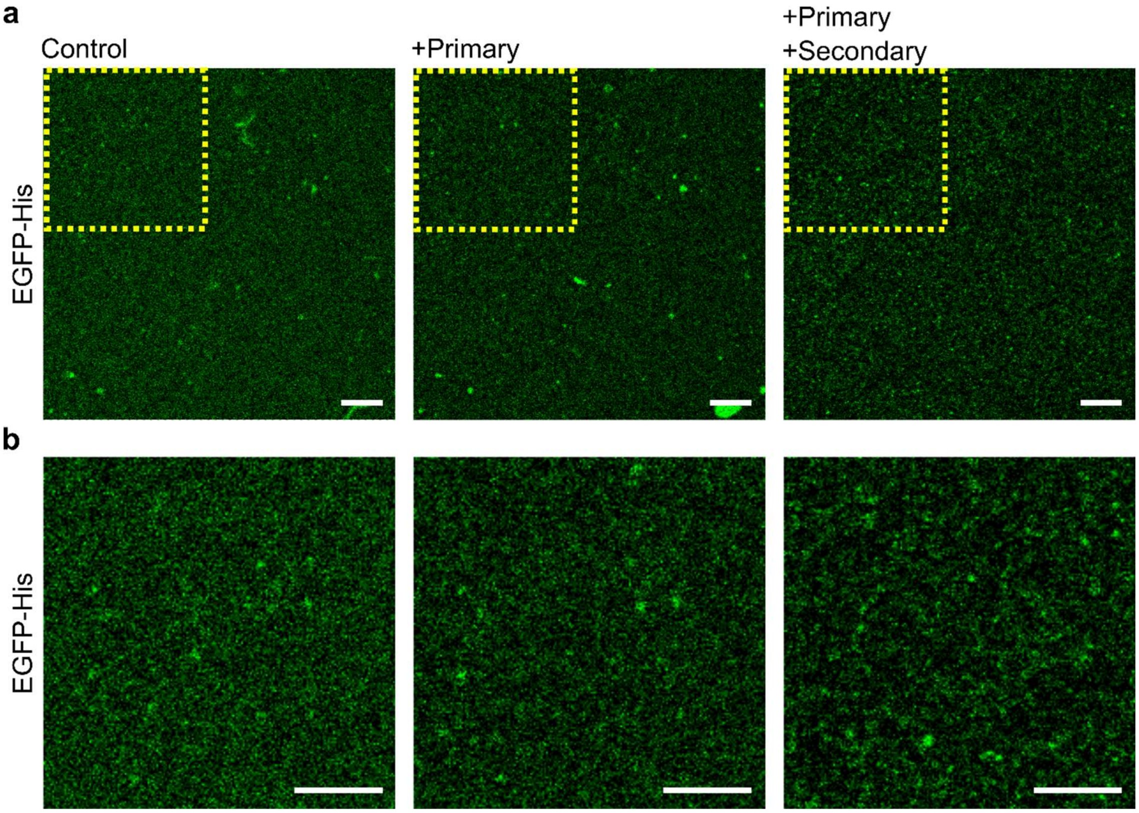

Supplementary Figure 10: Confocal Images of EGFP-His tagged bilayers. **a.** Overview images. The images are magnification of the image in Figure 2 panel b. The yellow boxes indicate the regions for the zoom-ins in panel **b.** All scalebars are 5 µm. Representative images from >3 independent experiments.

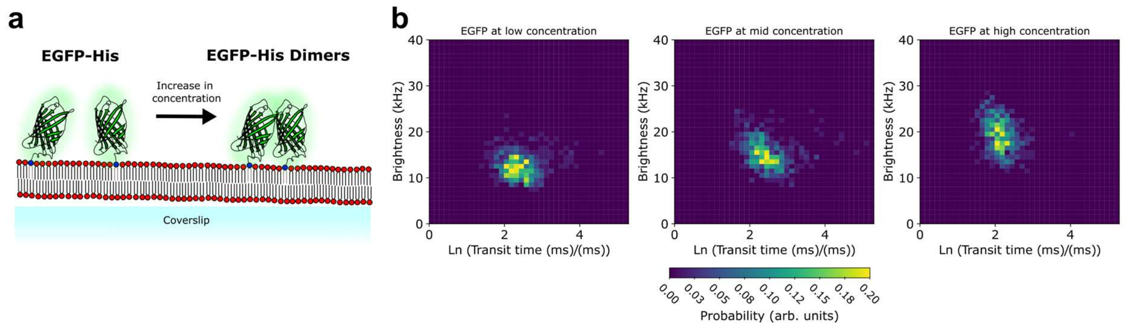

Supplementary Figure 11: Concentration dependence of EGFP-His brightness on the bilayer. a. Cartoon indicating concentration dependent oligomerisation of EGFP-His (EGFP visualised as green beta barrel inspired by PDB 1f0b[1]). **b.** BTS histograms from measurements of EGFP-His on nickelylated bilayers. The bilayers were incubated with EGFP-His at different concentrations (low 20 pM, mid 200 pM, and high 2 nM) and extensively washed before data were acquired. p-values from permutation test: high vs low < 0.01, other comparisons (high vs mid, mid vs low) are not significantly different.

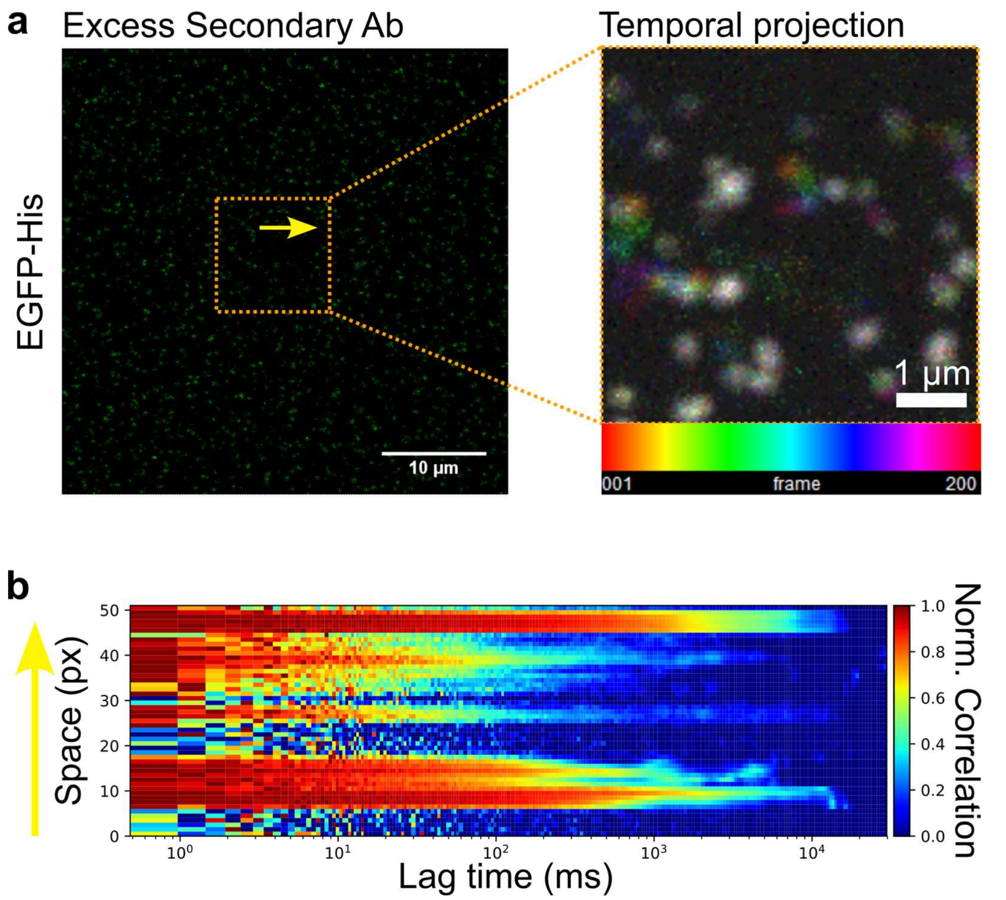

Supplementary Figure 12: Excess of secondary antibody leads to immobilisation. a Image and temporal projection of an imaging stack of EGFP-His oligomerised on an SLB by anti-GFP antibody and secondary antibody (122 ms per frame; 24.4 s total acquisition time; white particles are immobile on this time scale). **b** sFCS carpet from a measurement of clustered EGFP (sFCS raw data given as normalised correlation, from red to blue, over space). The yellow arrow in **a,b** indicates the sFCS line sampling over a few clusters. The sFCS carpet shows overwhelmingly signal from photo-bleaching or no correlation.

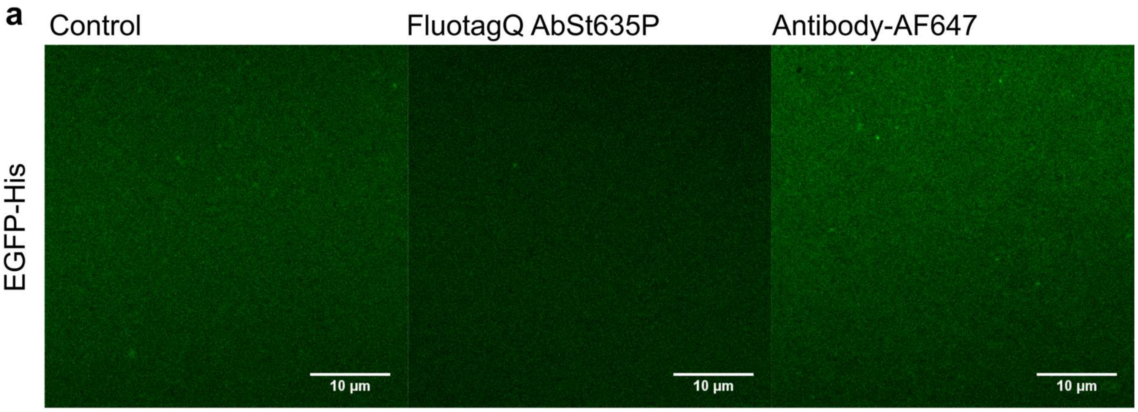

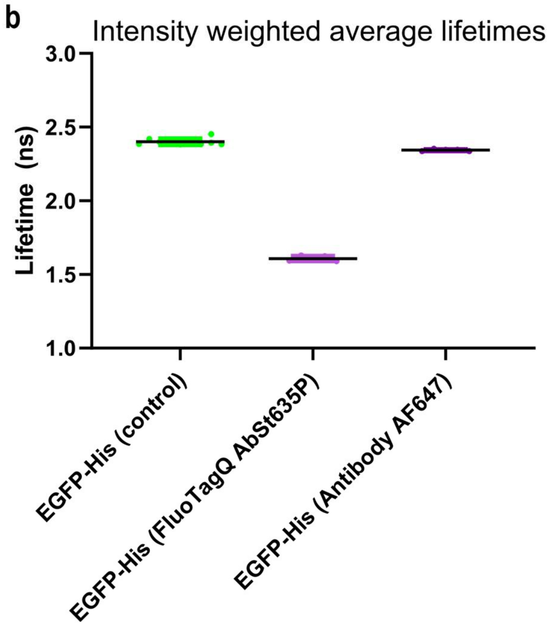

Supplementary Figure 13: Reduced brightness and lifetime of EGFP-His when bound to FluoTagQ nanobody. a Representative confocal image of SLBs doped with EGFP-His (control, left) and incubated with FluoTagQ-AbberiorSTAR 635P (middle) or anti-GFP antibody labelled with AF647 (right) from three independent acquisitions. **b** Intensity weighted average fluorescence lifetimes for EGFP-His and EGFP-His incubated with nanobody or antibody. Every dot represents one lifetime (FLIM) measurement. Black horizontal bar is mean value.

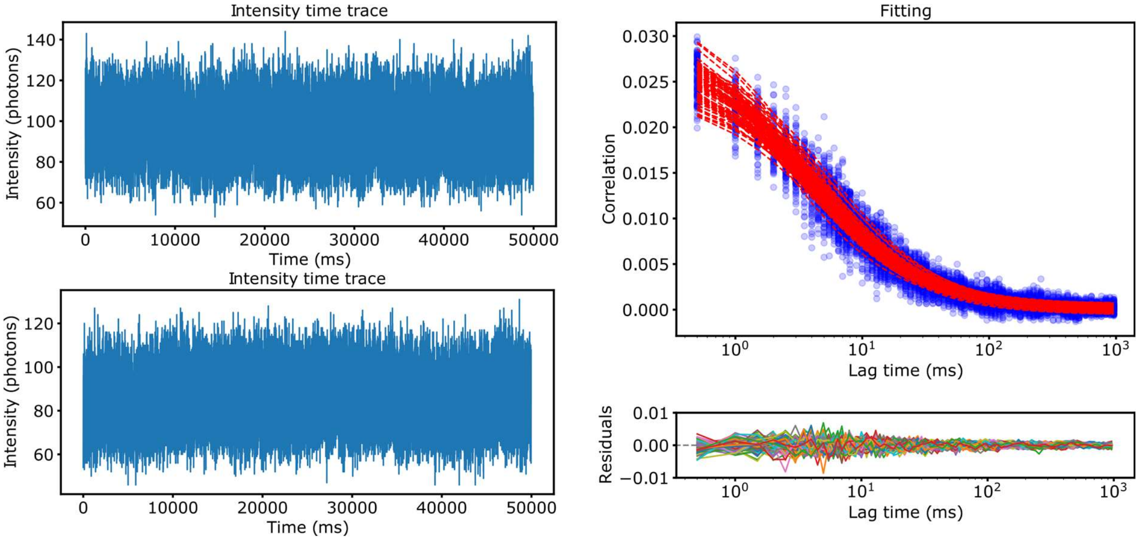

Supplementary Figure 14: Raw intensity traces and autocorrelation curve fitting for the AF488-His experiments. Shown are 2 sFCS measurements of 52 pixels each as intensity traces and fitting all resulting autocorrelation curves per pixel with a 2D diffusion model.

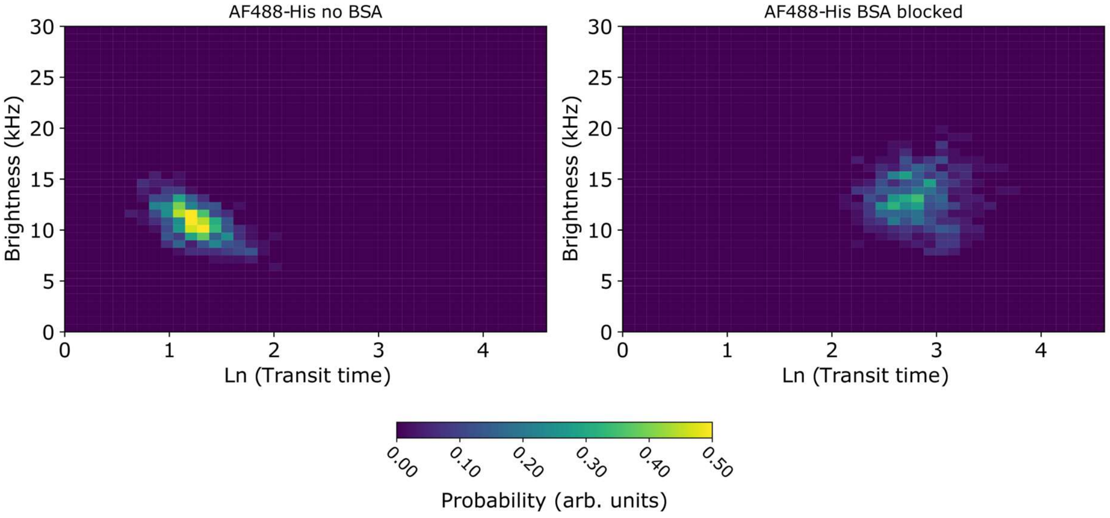

Supplementary Figure 15: Effect of BSA blocking on AF488-His decorated SLBs. Left panel shows the BTS data of AF488-His on an SLB which was not blocked with BSA nor washed with HSA containing HBS washing buffer. Right panel shows BTS data from an SLB that was blocked with BSA during sample preparation (same data as in main text Fig. 2j).

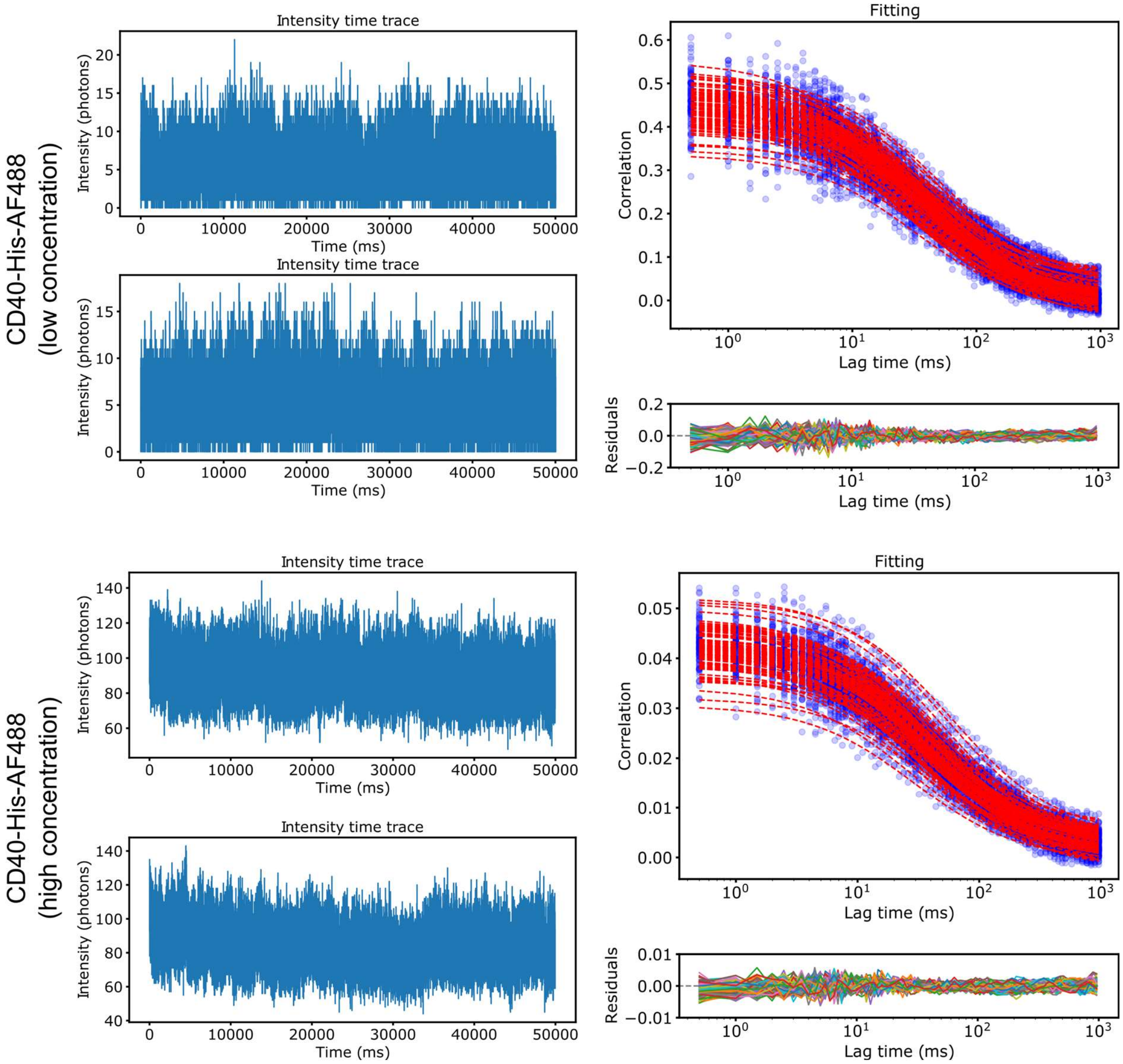

Supplementary Figure 16: Raw intensity traces and autocorrelation curve fitting for CD40-His-AF488 *in vitro* experiments. Shown are 2 sFCS measurements of 52 pixels each as intensity traces and fitting all resulting autocorrelation curves per pixel with a 2D diffusion model for CD40-His-AF488 at low concentration (25 molecules/µm^2, top panels) and high concentration (175 molecules/µm^2, bottom panels).

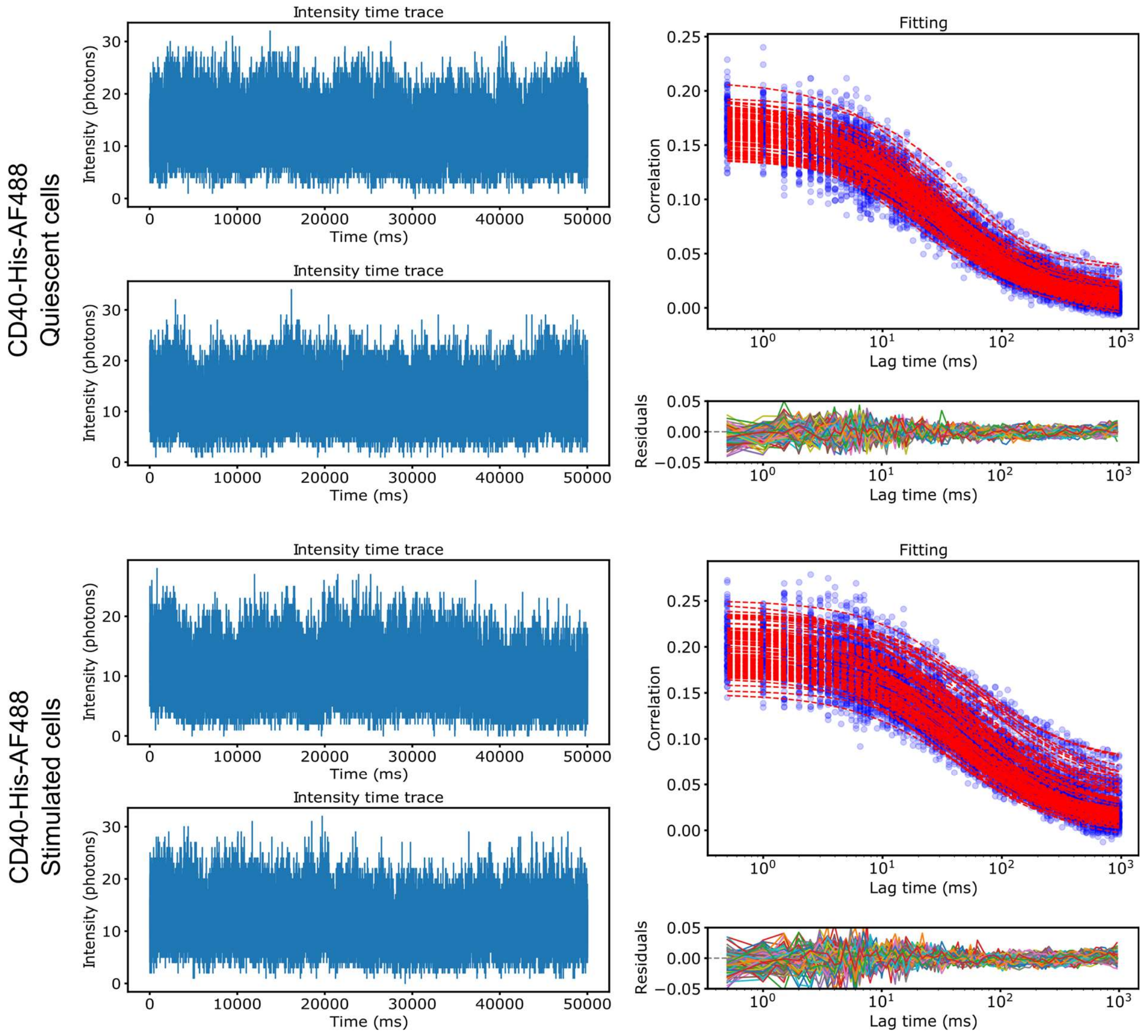

Supplementary Figure 17: Raw intensity traces and autocorrelation curve fitting for CD40-His-AF488 in presence of quiescent or stimulated cells. Shown are two sFCS measurements of 52 pixels each as intensity traces and fitting all resulting autocorrelation curves per pixel with a 2D diffusion model for CD40-His-AF488 in presence of quiescent cells (top panels) and stimulated cells (bottom panels).

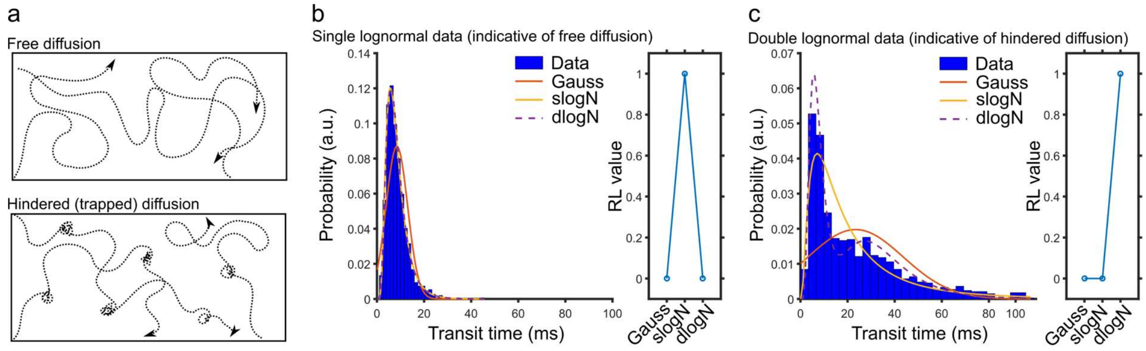

Supplementary Figure 18: Statistical Analysis of sFCS data: diffusion modalities. a: Cartoon depicting the molecular tracks of molecules undergoing free (top panel) and hindered/trapped diffusion (bottom panel). Nano-scale interactions cause transient halts in the diffusion path which might be indicative of molecular binding events. **b:** Statistical analysis of synthetic single lognormal data and synthetic double lognormal data **c**: Applying a Maximum Likelihood Estimation to the data allows to calculate a relative likelihood value (RL value) indicating which model (Gaussian normal distribution, single lognormal or double lognormal model) represents the data best (RL value = 1). Using this strategy on sFCS data enables to uncover hindered diffusion as freely diffusing molecules cause a transit time distribution best described by a single lognormal model. In contrast, the sFCS transit time histograms are best described by a double lognormal model when the underlying diffusion is hindered due to presence of nano-scale interactions.

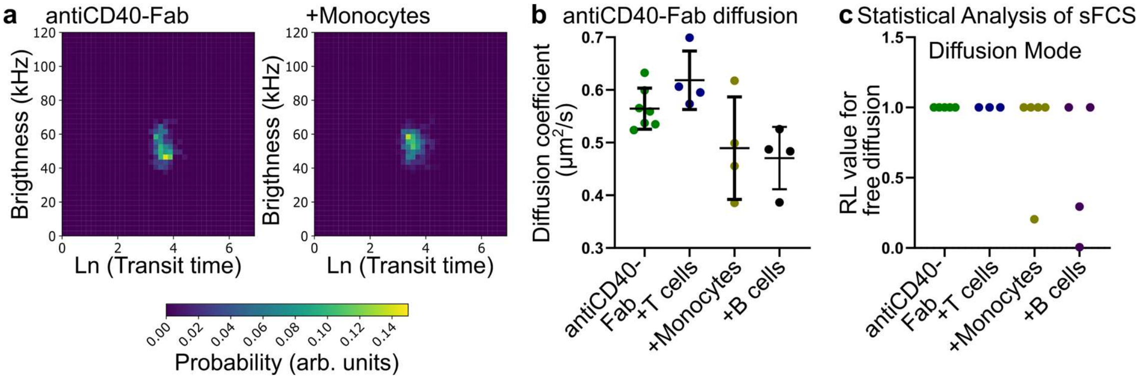

Supplementary Figure 19: sFCS acquisitions of antiCD40-Fab indicate slowed-down and hindered diffusion on B cells and monocytes. a: BTS plots for measurements on antiCD40-Fab labelled with AF647 and bound to an SLB (left) and interacting with monocytes (right). **b:** Diffusion coefficients extracted as population average from sFCS acquisitions on SLBs with antiCD40-Fab-AF647 (data also shown in Figure 3j in the main text, every dot represents >600 individual FCS curves pooled for each replicate, different donors for conditions with cells; n=7 for antiCD40-Fab control, n=4 for addition of T cells, n=4 for addition of B cells, and n=4 for addition of monocytes). Measurements on SLBs incubated with monocytes show a large variance. Error bars are standard deviation and horizontal lines are mean values **c:** Statistical analysis of sFCS data to reveal changes in diffusion mode. antiCD40-Fab alone and incubated with T cells shows free diffusion (for all repetitions relative likelihood value for free diffusion is 1). Hindrance in diffusion (interactions, RL values for free diffusion lower than 1) shows for monocytes and more pronounced for B cells. Primary cell data were acquired from 4 different donors. For every point in b,c hundreds of individual sFCS curves were integrated to show population level behaviour for one specific experiment.

Supplementary Table 1: Overview of methods to measure diffusion and/or oligomerisation of fluorescently labelled molecules. This is a non-exhaustive list indicating some of the most popular methods. We are comparing them with regards to working principles and application to measuring diffusion and oligomerisation and to give a sense where the BTS approach fits in.

Technique	Key-References	What does it do?	Method	Variations/ Improvements	Can it measure diffusion?	Can it be used to look at oligomerisation	Can it determine fractions of different oligomers?	How does it deal with a mixture of oligomers and monomers?
**FCS**	[2–4]	Autocorrelation of intensity trace and fitting to appropriate model	Single-point intensity trace	STED-FCS, sv-FCS, FLCS	Yes	Yes	Yes (but with limitations as higher order cumulants and correlations need to be used requiring large photon statistics/acquisition times or pure monomer and pure oligomer solutions need to be available)	N/A
**FIDA**	[5,6]	Statistical analysis of intensity probability distributions	Single-point intensity trace	2D-FIDA	Yes (when combined with FCS)	Yes	Yes	At single molecule concentrations: Provides identity and

								fractions of oligomers.<br><br>At higher concentration: Provides an average oligomer size at single point
**PCH**	[7,8]	Analysis of intensity by temporal binning	Single-point intensity trace		No	Yes	Yes	At single molecule concentrations: Provides identity and fractions of oligomers.<br><br>At higher concentration: Provides an average oligomer size at single point
**Cumulant Analysis**	[9]	Examines the autocorrelation function	Single-point intensity trace		Yes (combination with FCS)	Yes	Yes	Provides identity and fractions of oligomers at single point.
**N&B**	[10,11]	Moment analysis per pixel	Image time series based	eN&B	No	Yes	No – not in a single pixel	Provides average oligomeric state per pixel.

								Can provide fractions for spatially segregated oligomers
**RICS**	[12]	Spatio-temporal correlation (scanning method)	Image time series based	Local RICS	Yes (self-calibrated)	No	No	N/A
**STICS**	[13]	Spatio-temporal correlation (camera based)	Image time series based		Yes	No	No	N/A
**ICS**	[14,15]	Spatial correlation	Image time series based		No	Yes	No	Provides average oligomer size per pixel
**Imaging FCS**	[16,17]	Pixel-wise temporal correlation	Image time series based	SPIM-FCS	yes	Yes	No	Provides average oligomer size per pixel
**iMSD**	[18]	Spatio-temporal correlation and fitting of a Gaussian	Image time series based		Yes	No	No	N/A
**pCOMB**	[19]	Pair correlation analysis of molecular brightness	Confocal line scan (kymograph) based	cpCOMB	Yes	Yes	Yes (when spatially segregated)	Provides weighted average of oligomeric state per pixel.

								Can provide fractions for spatially segregated oligomers or resolve the oligomer mixture if their translocation times are different.
**SpIDA**	[20]	Intensity distribution analysis of pixels from sub-region of an image	Image intensity distribution based		No	Yes	Yes (when spatially segregated)	Provides average oligomer size per analysed ROI
**2D-FIF**	[21]	Intensity distribution analysis of pixels from sub-region of an image	Image intensity distribution based		No	Yes	Yes	Provides distribution of oligomeric states for every ROI<br><br>Estimates one average brightness for a pixel within a ROI
**SPT**	[22,23]	Single molecule brightness			Yes	No	No	N/A

**TOCCSL**	[24]	Single molecule brightness after bleaching	Image time series + FRAP based		Yes (combined with SPT)	Yes	Yes	Provides fractions of oligomers for analysed (bleached) ROI.
**FRET**	[25]	Energy transfer between a donor and an acceptor pair. Single molecule Fluorescence lifetimes	Single molecule fluorescence lifetime and intensity changes due to energy transfer between donor and acceptor molecules upon dimerization / oligomerization	Three-color FRET, FRET-FLIM	Yes (if combined with FLCS)	Yes	No (prior knowledge of oligomer makeup necessary for fitting lifetime component)	Limited to analysis of monomers and dimers (for two-colour FRET) and trimers (for three colour FRET)
**Fluorescence Anisotropy**	[26,27]	Polarized excitation + Polarized emission detection	Homo-FRET + fluorescence anisotropy based		No	Yes	Yes (when spatially segregated)	Provides average state for analysed region
**Fluorescence Antibunching / Photon**	[28–30]	Pulsed laser excitation + Time-Correlated Single Photon Counting	Nanosecond two photon correlation based		Yes (in solution when combined with FCS analysis)	Yes	Yes (when spatially segregated	Provides average oligomeric state per pixel

**coincidence imaging**								
**Photobleaching step analysis**	[31,32]	Widefield Imaging + Counting bleaching steps	Image time series based		No	Yes	Yes (at single molecule concentrations when spatially segregated)	Can provide fractions of oligomers for analysed region at single molecule concentrations
**BTS**	This work	Autocorrelation of intensity traces and histogram analysis of fitting parameters	Confocal line scan (kymograph) based		Yes	Yes	Yes (when spatially segregated)	Provides weighted average of oligomeric states at every pixel and the oligomeric state distribution for all sampled areas.

Supplementary Table 2: Diffusion coefficients and corresponding transit times as well as logarithm thereof for the simulated data in Figure 1 and S1,2. For the simulations a Gaussian observation spot with 240 nm FWHM has been assumed.

$D/\tau_{D,}$	Diffusion coefficient D ($\mu m^2/s$)	Transit time τ_D (ms)	Ln(τ_D)
1	0.01	1038.74	6.94
2	0.1	103.87	4.64
3	0.5	20.77	3.03
4	1	10.39	2.34
5	5	2.08	0.73
6	10	1.04	0.04

Supplementary Table 3: Summary of the EGFP-His data. Mean and standard deviation of diffusion coefficients and transit times as well as brightness for the data in Figure 2.

Condition	Diffusion coefficient D ($\mu m^2/s$)	Transit time τ_D (ms)	cpm (kHz)
EGFP-His (control)	2.3 (± 0.5)	5.9 (± 1.3)	13.7 (± 2.3)
EGFP-His (antibody)	0.6 (± 0.1)	20.9 (± 4.1)	20.9 ((± 2.8)
EGFP-His (antibody and secondary)	0.2 (± 0.1)	65.3 (± 29.4)	33.3 (± 4.6)
EGFP-His (control for nanobody)	3.0 (± 0.5)	4.7 (± 0.8)	14.9 (± 1.6)
EGFP-His (FluoTagQ nanobody)	2.6 (± 0.6)	5.5 (± 1.4)	8.3 ((± 1.6)

Supplementary Table 4: Summary of the CD40-His data *in vitro*. Mean and standard deviation of diffusion coefficients and transit times as well as brightness for the data in Figure 2.

Condition	Diffusion coefficient D ($\mu m^2/s$)	Transit time τ_D (ms)	cpm (kHz)
AF488-His_6 (monomeric control – no BSA blocking)	3.4 (± 0.7)	4.1 (± 1.0)	11.8 (± 1.6)
AF488-His_6 (monomeric control – BSA blocking)	0.8 (± 0.2)	17.2 (± 4.9)	13.1 (± 2.3)
CD40 (low density)	0.3 (± 0.1)	45.0 (± 14.6)	10.6 (± 1.7)
CD40 (mid density)	0.3 (± 0.1)	49.4 (± 12.3)	16.8 (± 1.8)
CD40 (high density)	0.3 (± 0.1)	46.4 (± 13.1)	17.2 (± 1.7)
CD40 (low density, control for interaction with antibodies)	0.3 (± 0.1)	41.8 (± 12.5)	9.1 (± 1.3)
CD40 with 5C3 antibody	0.2 (± 0.1)	83.2 (±36.8)	12.8 (±2.3)
CD40 with HB14 antibody	0.2 (± 0.1)	90.2 (± 36.3)	12.5 (±1.9)
CD40 with recombinant CD40L	0.1 (± 0.03)	178.0 (± 98.5)	14.5 (±2.9)

Table 5: Summary of the CD40-His data on bilayers interacting with primary T-cells. Mean and standard deviation of diffusion coefficients and transit times as well as brightness for the data in Figure 3.

Condition	Diffusion coefficient D ($\mu m^2/s$)	Transit time τ_D (ms)	cpm (kHz)
CD40 (plain SLB without cells at 37 C in presence of ICAM and antiCD3 fab)	0.5 (± 0.1)	27.4 (±9.2)	9.9 (±1.7)
CD40 (quiescent cells interacting with SLB)	0.4 (±0.1)	37.0 (±12.7)	9.1 (±1.1)
CD40 (stimulated cells interacting with SLB)	0.2 (±0.1)	108.1 (±220.3)	9.4 (±6.8)

Supplementary Table 6: Pairwise comparisons of BTS histograms and corresponding p-values using the permutation method (20% of the data have been used for bootstrapping 10,000 iterations)

Figure	Panel	Condition1	Condition2	p-Value
2	c	Control	Anti-GFP primary antibody	1.00E-04
2	c	Anti-GFP primary antibody	Secondary antibody	1.00E-04
2	c	Control	Secondary antibody	1.00E-04
2	g	Control	Anti-GFP nanobody	1.00E-04
2	k	Low CD40 concentration	Mid CD40 concentration	1.00E-04
2	k	Mid CD40 concentration	High CD40 concentration	1
2	k	Low CD40 concentration	High CD40 concentration	1.00E-04
2	l,m	CD40 control	5C3 anti-CD40-antibody	1.00E-04
2	l,m	CD40 control	HB14 anti-CD40-antibody	1.00E-04
2	l,m	5C3 anti-CD40-antibody	HB14 anti-CD40-antibody	1
2	l,m	CD40 control	CD40L	1.00E-04
2	l,m	5C3 anti-CD40-antibody	CD40L	0.011
2	l,m	HB14 anti-CD40-antibody	CD40L	0.100
3	g	CD40 control	Quiescent Cells	0.003
3	g	CD40 control	Stimulated Cells	0.15
3	g	Quiescent Cells	Stimulated Cells	0.99

Supplementary Table 7: Relative likelihood (RL) values for sFCS measurements on CD40-His-AF488. The RL value is given for the likelihood that the sFCS data distribution originated from measuring freely diffusing particles. Every entry in the table represents a set of measurements (ie. the RL value for one cell in the table is obtained from comparing different models for >10 sFCS measurements of one condition with multiple measurements).

CD40 control (no cells)	CD40 with quiescent cells	CD40 with stimulated cells
1	1	8.12e-117
1	1	3.3e-007
1	3.98e-021	1
1	1	3.7e-011
	1.61e-014	1.95e-053
	2.73e-009	7.25e-008
		1

Supplementary References

1. Wachter, R. M., Yarbrough, D., Kallio, K. & Remington, S. J. Crystallographic and energetic analysis of binding of selected anions to the yellow variants of green fluorescent protein. *J. Mol. Biol.* **301**, 157–171 (2000).
2. Magde, D., Elson, E. & Webb, W. W. Thermodynamic Fluctuations in a Reacting System---Measurement by Fluorescence Correlation Spectroscopy. *Phys. Rev. Lett.* **29**, 705–708 (1972).
3. Elson, E. L. Fluorescence Correlation Spectroscopy: Past, Present, Future. *Biophys. J.* **101**, 2855–2870 (2011).
4. Sankaran, J. & Wohland, T. Current capabilities and future perspectives of FCS: super-resolution microscopy, machine learning, and in vivo applications. *Commun. Biol.* **6**, 1–11 (2023).
5. Kask, P., Palo, K., Ullmann, D. & Gall, K. Fluorescence-intensity distribution analysis and its application in biomolecular detection technology. *Proc. Natl. Acad. Sci.* **96**, 13756–13761 (1999).
6. Palo, K. *et al.* Fluorescence intensity and lifetime distribution analysis: toward higher accuracy in fluorescence fluctuation spectroscopy. *Biophys. J.* **83**, 605–618 (2002).
7. Chen, Y., Müller, J. D., So, P. T. C. & Gratton, E. The Photon Counting Histogram in Fluorescence Fluctuation Spectroscopy. *Biophys. J.* **77**, 553–567 (1999).
8. Müller, J. D., Chen, Y. & Gratton, E. Resolving heterogeneity on the single molecular level with the photon-counting histogram. *Biophys. J.* **78**, 474–486 (2000).
9. Müller, J. D. Cumulant Analysis in Fluorescence Fluctuation Spectroscopy. *Biophys. J.* **86**, 3981–3992 (2004).
10. Digman, M. A., Dalal, R., Horwitz, A. F. & Gratton, E. Mapping the Number of Molecules and Brightness in the Laser Scanning Microscope. *Biophys. J.* **94**, 2320 (2008).
11. Cutrale, F. *et al.* Using enhanced number and brightness to measure protein oligomerization dynamics in live cells. *Nat. Protoc.* **14**, 616–638 (2019).
12. Digman, M. A. *et al.* Fluctuation Correlation Spectroscopy with a Laser-Scanning Microscope: Exploiting the Hidden Time Structure. *Biophys. J.* **88**, L33–L36 (2005).

13. Hebert, B., Costantino, S. & Wiseman, P. W. Spatiotemporal Image Correlation Spectroscopy (STICS) Theory, Verification, and Application to Protein Velocity Mapping in Living CHO Cells. *Biophys. J.* **88**, 3601–3614 (2005).

14. Kolin, D. L. & Wiseman, P. W. Advances in image correlation spectroscopy: measuring number densities, aggregation states, and dynamics of fluorescently labeled macromolecules in cells. *Cell Biochem. Biophys.* **49**, 141–164 (2007).

15. Petersen, N. O., Höddelius, P. L., Wiseman, P. W., Seger, O. & Magnusson, K. E. Quantitation of membrane receptor distributions by image correlation spectroscopy: concept and application. *Biophys. J.* **65**, 1135–1146 (1993).

16. Burkhardt, M. & Schwille, P. Electron multiplying CCD based detection for spatially resolved fluorescence correlation spectroscopy. *Opt. Express* **14**, 5013–5020 (2006).

17. Kannan, B. *et al.* Electron Multiplying Charge-Coupled Device Camera Based Fluorescence Correlation Spectroscopy. *Anal. Chem.* **78**, 3444–3451 (2006).

18. Di Rienzo, C., Gratton, E., Beltram, F. & Cardarelli, F. Fast spatiotemporal correlation spectroscopy to determine protein lateral diffusion laws in live cell membranes. *Proc. Natl. Acad. Sci.* **110**, 12307–12312 (2013).

19. Hinde, E. *et al.* Quantifying the dynamics of the oligomeric transcription factor STAT3 by pair correlation of molecular brightness. *Nat. Commun.* **7**, 11047 (2016).

20. Godin, A. G. *et al.* Revealing protein oligomerization and densities in situ using spatial intensity distribution analysis. *Proc. Natl. Acad. Sci.* **108**, 7010–7015 (2011).

21. Stoneman, M. R. *et al.* A general method to quantify ligand-driven oligomerization from fluorescence-based images. *Nat. Methods* **16**, 493–496 (2019).

22. Jaqaman, K. *et al.* Robust single-particle tracking in live-cell time-lapse sequences. *Nat. Methods* **5**, 695–702 (2008).

23. Kusumi, A. *et al.* Paradigm shift of the plasma membrane concept from the two-dimensional continuum fluid to the partitioned fluid: high-speed single-molecule tracking of membrane molecules. *Annu. Rev. Biophys. Biomol. Struct.* **34**, 351–378 (2005).
24. Moertelmaier, M., Brameshuber, M., Linimeier, M., Schütz, G. J. & Stockinger, H. Thinning out clusters while conserving stoichiometry of labeling. *Appl. Phys. Lett.* **87**, 263903 (2005).
25. Chung, H. S. *et al.* Oligomerization of the tetramerization domain of p53 probed by two- and three-color single-molecule FRET. *Proc. Natl. Acad. Sci.* **114**, E6812–E6821 (2017).
26. Chan, F. T. S., Kaminski, C. F. & Kaminski Schierle, G. S. HomoFRET Fluorescence Anisotropy Imaging as a Tool to Study Molecular Self-Assembly in Live Cells. *ChemPhysChem* **12**, 500–509 (2011).
27. Heckmeier, P. J. *et al.* Determining the Stoichiometry of Small Protein Oligomers Using Steady-State Fluorescence Anisotropy. *Biophys. J.* **119**, 99–114 (2020).
28. Grußmayer, K. S. & Herten, D.-P. Photon Antibunching in Single Molecule Fluorescence Spectroscopy. in *Advanced Photon Counting: Applications, Methods, Instrumentation* (eds. Kapusta, P., Wahl, M. & Erdmann, R.) 159–190 (Springer International Publishing, Cham, 2015). doi:10.1007/4243_2014_71.
29. Sýkora, J. *et al.* Exploring Fluorescence Antibunching in Solution To Determine the Stoichiometry of Molecular Complexes. *Anal. Chem.* **79**, 4040–4049 (2007).
30. Kurz, A. *et al.* Counting Fluorescent Dye Molecules on DNA Origami by Means of Photon Statistics. *Small* **9**, 4061–4068 (2013).
31. Hummert, J. *et al.* Photobleaching step analysis for robust determination of protein complex stoichiometries. *Mol. Biol. Cell* **32**, ar35 (2021).
32. Grußmayer, K. S., Kurz, A. & Herten, D.-P. Single-Molecule Studies on the Label Number Distribution of Fluorescent Markers. *ChemPhysChem* **15**, 734–742 (2014).
