# Supplementary material for: Quantifying biomolecular organisation in membranes with brightness-transit statistics
Source: Nat Commun. 2024 Aug 17;15:7082. doi: 10.1038/s41467-024-51435-1 (PMC11329664; doi:10.1038/s41467-024-51435-1)
Supplement: Supplementary file 2 — Reporting Summary [file 41467_2024_51435_MOESM2_ESM.pdf]

nature portfolio

Corresponding author(s): Marco Fritzsche, Falk Schneider

Last updated by author(s): Jul 12, 2024

Reporting Summary

Nature Portfolio wishes to improve the reproducibility of the work that we publish. This form provides structure for consistency and transparency in reporting. For further information on Nature Portfolio policies, see our Editorial Policies and the Editorial Policy Checklist.

Statistics

For all statistical analyses, confirm that the following items are present in the figure legend, table legend, main text, or Methods section.

n/a	Confirmed	
☐	☒	The exact sample size (*n*) for each experimental group/condition, given as a discrete number and unit of measurement
☐	☒	A statement on whether measurements were taken from distinct samples or whether the same sample was measured repeatedly
☐	☒	The statistical test(s) used AND whether they are one- or two-sided<br>*Only common tests should be described solely by name; describe more complex techniques in the Methods section.*
☒	☐	A description of all covariates tested
☐	☒	A description of any assumptions or corrections, such as tests of normality and adjustment for multiple comparisons
☐	☒	A full description of the statistical parameters including central tendency (e.g. means) or other basic estimates (e.g. regression coefficient) AND variation (e.g. standard deviation) or associated estimates of uncertainty (e.g. confidence intervals)
☐	☒	For null hypothesis testing, the test statistic (e.g. *F*, *t*, *r*) with confidence intervals, effect sizes, degrees of freedom and *P* value noted<br>*Give P values as exact values whenever suitable.*
☐	☒	For Bayesian analysis, information on the choice of priors and Markov chain Monte Carlo settings
☒	☐	For hierarchical and complex designs, identification of the appropriate level for tests and full reporting of outcomes
☒	☐	Estimates of effect sizes (e.g. Cohen's *d*, Pearson's *r*), indicating how they were calculated

Our web collection on statistics for biologists contains articles on many of the points above.

Software and code

Policy information about availability of computer code

Data collection	The Zeiss LSM 780 was operated using the provided Zen Black 2011 (service pack 7) software and the Zeiss 980 was operated using Zen Blue. Data were saved as .lsm5 or .czi files.
Data analysis	Images were processed in FIJI (ImageJ 2.0.0-rc-59/1.53k; Java 1.8.0_66). Point fluctuation data were processed with FoCuS_point software (1_16_203, available at https://github.com/dwaithe/FCS_point_correlator). Scanning fluctuation data were processed with FoCuS_Scan (1_14_74, available at https://github.com/dwaithe/FCS_scanning_correlator) in conjunction with custom code written for this study available at https://github.com/Faldalf/sFCS_BTS. The Python scripts (Python 3.7.7) were edited and run in the anaconda native IDE Spyder (version 4.1.5). Simple plotting and statistical tests (ANOVA, t-test) were performed in GraphPad Prism (version 10.1.2 (324)).

For manuscripts utilizing custom algorithms or software that are central to the research but not yet described in published literature, software must be made available to editors and reviewers. We strongly encourage code deposition in a community repository (e.g. GitHub). See the Nature Portfolio guidelines for submitting code & software for further information.

Data

Policy information about availability of data

All manuscripts must include a data availability statement. This statement should provide the following information, where applicable:

- Accession codes, unique identifiers, or web links for publicly available datasets
- A description of any restrictions on data availability
- For clinical datasets or third party data, please ensure that the statement adheres to our policy

Exemplary sFCS fitting results and raw data are deposited on GITHUB (https://github.com/Faldalf/sFCS_BTS). All other raw data are available from the corresponding authors upon reasonable request.

Research involving human participants, their data, or biological material

Policy information about studies with human participants or human data. See also policy information about sex, gender (identity/presentation), and sexual orientation and race, ethnicity and racism.

Reporting on sex and gender	N/A
Reporting on race, ethnicity, or other socially relevant groupings	N/A
Population characteristics	N/A
Recruitment	N/A
Ethics oversight	N/A

Note that full information on the approval of the study protocol must also be provided in the manuscript.

Field-specific reporting

Please select the one below that is the best fit for your research. If you are not sure, read the appropriate sections before making your selection.

[x] Life sciences [ ] Behavioural & social sciences [ ] Ecological, evolutionary & environmental sciences

For a reference copy of the document with all sections, see nature.com/documents/nr-reporting-summary-flat.pdf

Life sciences study design

All studies must disclose on these points even when the disclosure is negative.

Sample size	No sample-size calculations were performed. For the number of simulations and acquisitions of experimental scanning FCS data we followed accepted standards in the field (e.g., PMID: 25410140, PMID: 30028588, PMID: 37106176). We simulated a set of 500 curves spread over ten independent simulations. For the experimental data, we pooled sFCS curves from different bilayers and >10 cells (if applicable) for every independent repeat.
Data exclusions	No data were excluded from this study.
Replication	All simulations and experiments were performed multiple times (>3) on different days and using different samples or cell preparations (as applicable). All attempts on replication were successful.
Randomization	Randomization is not relevant to this study in a traditional sense as there was no random assignment of participants or test subjects to different groups. Experimental conditions involving primary cells were repeated for all conditions on samples from different donors.
Blinding	Blinding was not possible as all samples were prepared and measured by the same individual. Analysis was performed using automated pipelines and model selection to combat any bias.

Reporting for specific materials, systems and methods

We require information from authors about some types of materials, experimental systems and methods used in many studies. Here, indicate whether each material, system or method listed is relevant to your study. If you are not sure if a list item applies to your research, read the appropriate section before selecting a response.

Materials & experimental systems

n/a	Involved in the study
	[x] Antibodies
[x]	[ ] Eukaryotic cell lines
[x]	[ ] Palaeontology and archaeology
[x]	[ ] Animals and other organisms
[x]	[ ] Clinical data
[x]	[ ] Dual use research of concern
[x]	[ ] Plants

Methods

n/a	Involved in the study
[x]	[ ] ChIP-seq
[x]	[ ] Flow cytometry
[x]	[ ] MRI-based neuroimaging

Antibodies

Antibodies used

GFP Alexa Fluor® 647-conjugated Antibody (R&D Systems)
https://www.rndsystems.com/products/gfp-alexa-fluor-647-conjugated-antibody-454505r_fab42402r
FAB42402R-100UG
Used 1:1000

FluotagQ labelled with AbberiorSTAR635P
https://nano-tag.com/products/fluotag-q-anti-gfp
Custom request from NanoTag
Used 1:1000

Purified Anti Human CD40 Antibody (Biolegend) Clone HB14
hhttps://www.biolegend.com/en-us/products/purified-anti-human-cd40-antibody-2361?GroupID=BLG11946
#313002
Used at 1 µg/mL (1:500 dilution of 0.5mg/mL stock) for in vitro oligomerisation experiments.

Purified Anti Human CD40 Antibody (Biolegend) Clone 5C3
https://www.biolegend.com/fr-fr/search-results/purified-anti-human-cd40-maxpar-ready-antibody-10144
#334325
Used at 1 µg/mL (1:1000 dilution of 1mg/mL stock) for in vitro oligomerisation experiments.

Goat anti-Mouse IgG2a (Fisher Scientific / Invitrogen)
https://www.fishersci.com/shop/products/alexa-fluor-647-goat-a/a21241Polyclonal/A-31571
A21241
Used 1:100 - 1:1000

Anti-CD40 Clone G28.5 (Absolute Antibody)
https://absoluteantibody.com/product/anti-cd40-g28-5/
Ab00129-10
Used 1:4000

Validation

Validations and references are provided on the supplier's homepages:
GFP Alexa Fluor® 647-conjugated Antibody (R&D Systems)
https://www.rndsystems.com/products/gfp-alexa-fluor-647-conjugated-antibody-454505r_fab42402r

FluotagQ labelled with AbberiorSTAR635P
https://nano-tag.com/products/fluotag-q-anti-gfp

Purified Anti Human CD40 Antibody (Biolegend) Clone HB14
hhttps://www.biolegend.com/en-us/products/purified-anti-human-cd40-antibody-2361?GroupID=BLG11946

Goat anti-Mouse IgG2a (Fisher Scientific / Invitrogen)
https://www.fishersci.com/shop/products/alexa-fluor-647-goat-a/a21241Polyclonal/A-31571

Purified Anti Human CD40 Antibody (Biolegend) Clone 5C3
https://www.biolegend.com/fr-fr/search-results/purified-anti-human-cd40-maxpar-ready-antibody-10144

Anti CD40 Antibody G28.5
https://absoluteantibody.com/product/anti-cd40-g28-5/

Plants

Seed stocks	N/A
Novel plant genotypes	N/A
Authentication	N/A
